# Severe Holoprosencephaly With Arhinencephaly in A Liveborn Neonate: A Case Report

**DOI:** 10.1002/ccr3.73103

**Published:** 2026-07-05

**Authors:** Sarah K. Kebbeh, Hamdi I. Nawfal

**Affiliations:** ^1^ Aleppo University Aleppo Syria

**Keywords:** alobar‐semilobar HPE, arhinencephaly, case report, craniofacial malformations, holoprosencephaly, prenatal diagnosis

## Abstract

Holoprosencephaly (HPE) is a rare congenital brain malformation resulting from incomplete separation of the cerebral hemispheres. It has an estimated prevalence of 1 in 10,000 live births. We report a case involving an 18‐year‐old primigravida at 28 weeks of gestation. Prenatal ultrasound revealed polyhydramnios, marked hydrocephalus, and absence of the cerebral and cerebellar hemispheres, with preservation of the brainstem. Cesarean delivery was performed, and the female neonate exhibited hypotelorism, arhinia, cleft lip and palate, and a single frontal bony plate (neonatal weight, head circumference, and Apgar scores were not available). The constellation of findings was consistent with severe holoprosencephaly within the alobar‐semilobar spectrum with associated arhinencephaly. This case highlights a rare presentation of severe HPE with associated arhinencephaly in a liveborn neonate, occurring in the absence of identifiable risk factors. It underscores the critical role of prenatal imaging in the diagnosis of midline anomalies and the need for thorough postnatal evaluation.

## Key Clinical Message

This case highlights a rare live birth with severe holoprosencephaly and arhinencephaly, absent known risk factors. It emphasizes the vital role of prenatal imaging in detecting severe midline anomalies and the importance of thorough postnatal evaluation.

## Graphical abstract

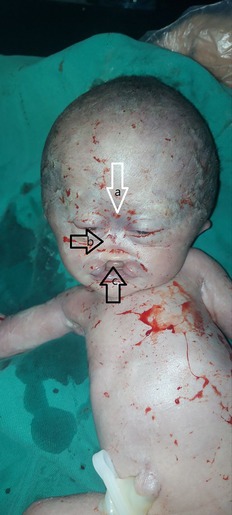

## 1 Introduction

Holoprosencephaly (HPE) is a congenital brain malformation resulting from incomplete division of the prosencephalon (embryonic forebrain) into two cerebral hemispheres, typically occurring between the third and fourth weeks of gestation [1]. HPE is classified into three types based on the degree of hemispheric separation: alobar (most severe), semilobar, and lobar (least severe) [1].

Alobar HPE, the most severe form, accounts for approximately two‐thirds of cases. In about 80% of affected fetuses, craniofacial anomalies are present. These range from severe deformities such as cyclopia, synophthalmia, and proboscis to milder features like microcephaly, hypotelorism, depressed nasal bridge, single maxillary central incisor, and midline cleft lip and palate [1].

Diagnosis is typically made prenatally using ultrasound and magnetic resonance imaging (MRI), particularly in severe cases. In milder forms, diagnosis may be based on postnatal clinical features and neuroimaging [1, 2, 3, 4].

The estimated prevalence of HPE is approximately 1 in 10,000 live births, but it is significantly more common in spontaneously aborted fetuses, with a prevalence of about 1 in 250, making it the most frequent forebrain malformation in humans [1].

## 2 Case History and Examination

We report the case of an 18‐year‐old primigravida who presented to the labor ward with preterm labor at 28 weeks of gestation.

Ultrasound examination revealed a female fetus with polyhydramnios, marked hydrocephalus, and absence of the cerebrum and cerebellum, with preservation of the brainstem. This was the first echography performed during pregnancy.

**FIGURE 1:**
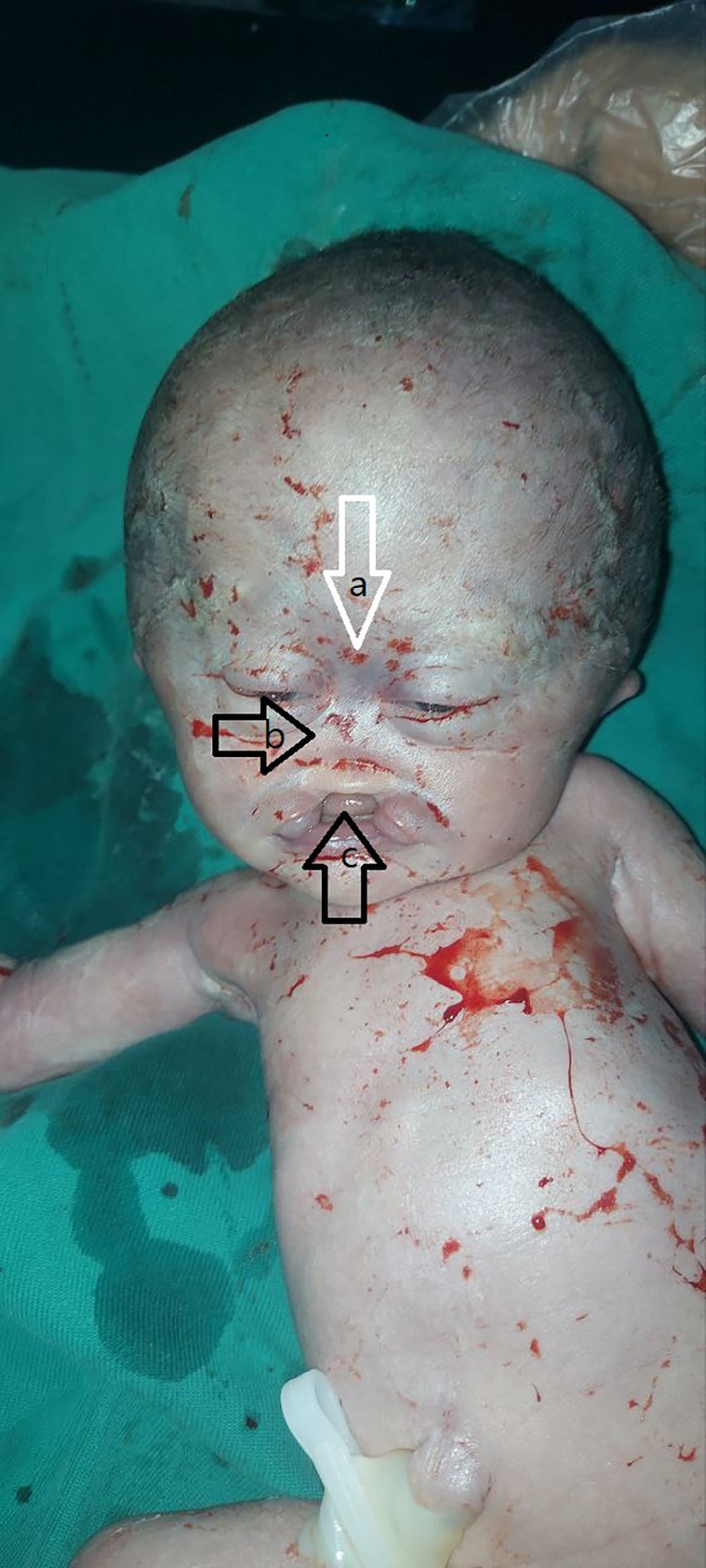
Physical examination revealed a‐hypotelorism, b‐arhinia, and c‐cleft lip and palate.

**FIGURE 2:**
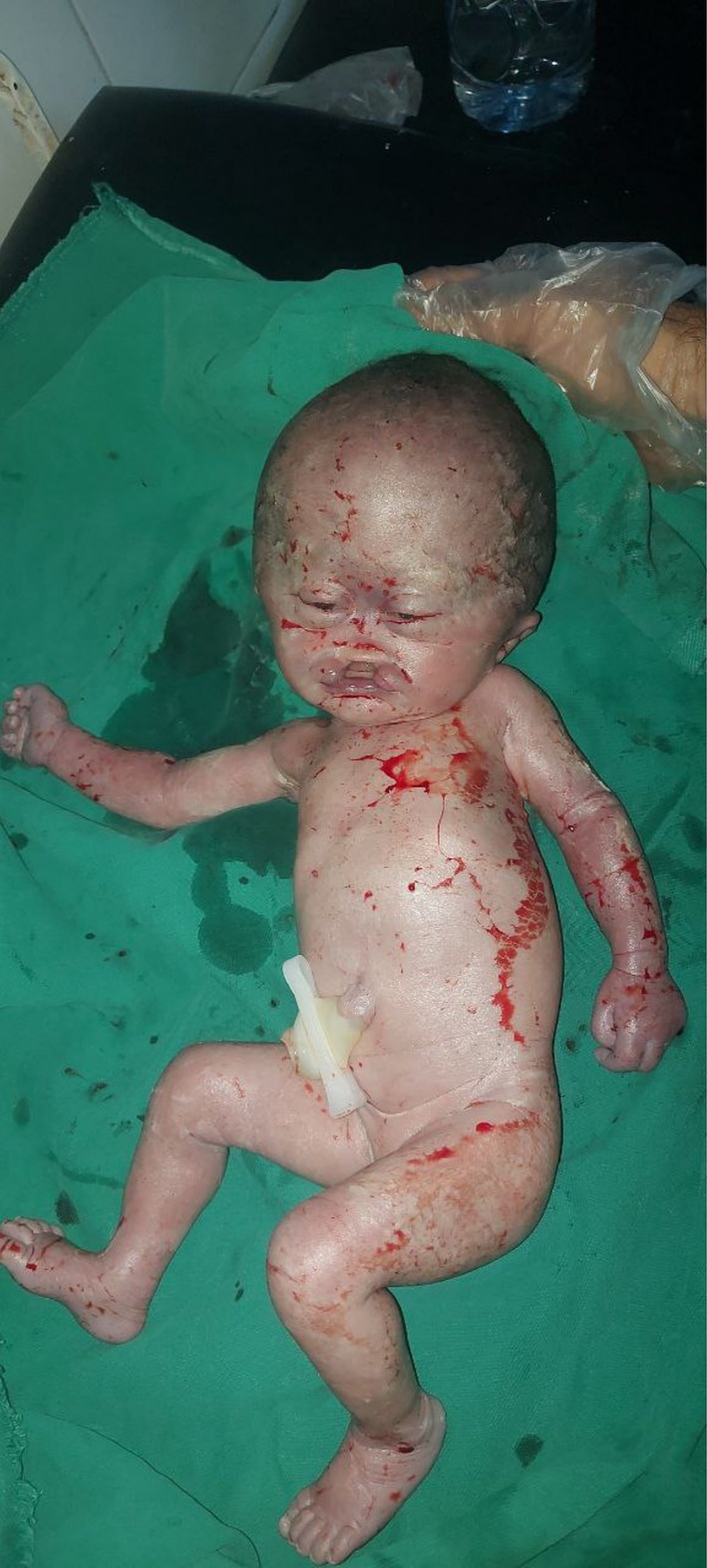
The extremities and the trunk were normal.

After informing parents about the prognosis of the expected diagnosis, a lower segment cesarean section was performed due to abnormal fetal presentation. Physical examination revealed hypotelorism, arhinia, and cleft lip and palate (Figure 1). A single frontal bony plate measuring approximately 2 × 2 cm^2^ was observed, with absence of the remaining cranial vault. All other organ systems were normal (Figure 2), and postnatal investigations, including karyotype analysis, showed no abnormalities. Additional Molecular tests were not performed due to their high costs.

Some neonatal anthropometric data, including weight, OFC, and Apgar score, were not documented in the medical record.

## 3 Differential Diagnosis, Investigations, and Treatmment

The constellation of craniofacial and neurological anomalies was most consistent with severe holoprosencephaly likely within the alobar‐semilobar spectrum with arhinencephaly, though severe anencephaly or hydranencephaly may also be considered in the differential diagnosis.

There was no consanguinity between the parents. The mother had no relevant medical history and reported no medication intake before or during pregnancy. The father had a history of haloperidol use (an antipsychotic medication) both 1 year before and during the pregnancy.

## 4 Outcome and Follow‐Up

The neonate died shortly after birth. Examination confirmed craniofacial anomalies with otherwise normal systemic findings. Karyotype analysis was normal, and no follow‐up was possible due to early neonatal demise.

## 5 Discussion

Holoprosencephaly (HPE) is a rare congenital brain malformation characterized by incomplete separation of the cerebral hemispheres, with a reported prevalence of approximately 1 in 10,000 live births. Most cases are incompatible with life, and up to 80% result in spontaneous abortion during early gestation [1]. The present case is unusual in that the neonate was born alive but died shortly after birth, representing a rare outcome in the clinical spectrum of HPE.

Clinically, the neonate exhibited severe craniofacial anomalies compatible with holoprosencephaly within the alobar–semilobar spectrum, including hypotelorism and midline facial cleft. Notably, arhinia was observed, along with absence of nasal bulbs—findings consistent with arhinencephaly, a rare and severe midline craniofacial defect that may accompany holoprosencephaly [1, 5, 6]. Postnatal MRI and CT, the diagnostic gold standard for HPE classification, were not feasible due to limited local resources and the neonate's rapidly deteriorating clinical condition. Newborn autopsy was not considered due to cultural reasons. The prenatal ultrasound findings, particularly the reported absence of the cerebral and cerebellar hemispheres with preservation of the brainstem, may be more suggestive of alobar holoprosencephaly according to conventional neuroimaging classifications. However, definitive classification was not possible because postnatal MRI, CT imaging, and autopsy were unavailable. Therefore, the term “alobar–semilobar spectrum” was adopted to reflect the diagnostic uncertainty while remaining consistent with the available prenatal and postnatal findings.

Important neonatal anthropometric parameters, including birth weight, occipitofrontal circumference, and Apgar scores, were unavailable because of incomplete documentation in the emergency clinical setting. This limitation restricted comprehensive phenotypic assessment and further characterization of disease severity.

The etiology of HPE is multifactorial and includes both genetic and non‐genetic causes. Genetic factors encompass both syndromic and non‐syndromic forms. Among the non‐syndromic cases, mutations in the Sonic Hedgehog (SHH) gene are most commonly implicated, often in association with midline craniofacial defects [7]. Syndromic forms of HPE are most frequently associated with trisomy 13 (Patau syndrome), followed by trisomy 18 and triploidy [8].

Previous case reports have described semilobar holoprosencephaly in association with maternal metabolic and hypertensive disorders. Maternal pregestational diabetes mellitus, particularly type 1 diabetes, has been recognized as a significant risk factor for holoprosencephaly, with reported cases of semilobar HPE occurring in diabetic pregnancies. Similarly, an association between semilobar holoprosencephaly and early‐onset preeclampsia has been described in isolated case reports [9, 10].

In contrast, the present case occurred in the absence of maternal diabetes, hypertensive disorders, or other identifiable maternal or environmental risk factors. This variability supports the concept that holoprosencephaly has a heterogeneous and multifactorial etiology and may arise even in pregnancies without recognized predisposing conditions.

Additional reports have documented severe forms of holoprosencephaly in live‐born neonates, often in association with chromosomal abnormalities such as trisomy 13 (Patau syndrome), where semilobar or alobar HPE may coexist with profound craniofacial anomalies. Furthermore, published cases describing severe HPE within the alobar–semilobar spectrum highlight the marked phenotypic variability and the diagnostic challenges encountered in the absence of advanced neuroradiological or pathological confirmation [11, 12]. These reports further contextualize the rarity of live‐born presentations and support the spectrum‐based diagnostic approach adopted in the present case.

Although the karyotype analysis was normal, nonsyndromic monogenic causes are well recognized in holoprosencephaly, most commonly involving genes such as SHH, ZIC2, SIX3, and TGIF, which play key roles in forebrain and midline development [8, 13]. Advanced genetic testing, including chromosomal microarray or targeted gene sequencing, was not performed in the present case due to limited availability and high cost.

Non‐genetic contributors include maternal metabolic conditions such as pre‐gestational diabetes mellitus, as well as folic acid deficiency during early pregnancy. Experimental models have identified several teratogens capable of disrupting forebrain development, including ethanol, salicylates, retinoic acid, and mycotoxins like ochratoxin A. Agents that interfere with Hedgehog signaling, such as cyclopamine, or that impair cholesterol biosynthesis have also been implicated. Environmental exposures such as tobacco smoke, charred meats, cannabis, and piperonyl butoxide (a commonly used pesticide) are additional potential risk factors [8].

In the current case, there was no history of consanguinity, familial genetic disorders, or maternal medical conditions. The mother did not report taking any medications before or during pregnancy. Karyotype analysis of the neonate revealed a normal chromosomal profile. The father had a history of haloperidol use; however, current evidence does not support paternal haloperidol exposure as a risk factor for congenital malformations.

## 6 Conclusion

This case presents a rare instance of severe holoprosencephaly within the alobar–semilobar spectrum with associated arhinencephaly in a liveborn neonate, with no identifiable genetic, maternal, or environmental risk factors. It underscores the complexity of HPE etiopathogenesis and the challenges of accurate classification in the absence of postnatal neuroimaging or autopsy. The findings emphasize the importance of thorough prenatal evaluation and postnatal investigation in cases of suspected midline anomalies. Furthermore, genetic counseling should be offered to the family in future pregnancies, particularly when advanced genetic testing is unavailable. Improved neonatal documentation in resource‐limited and crisis settings is also essential to facilitate accurate phenotypic characterization and clinical reporting.

This case report was prepared in accordance with CARE guidelines [14].

## Author Contributions

**Sarah K. Kebbeh:** resources, writing – review and editing. **Hamdi I. Nawfal:** supervision, writing – review and editing.

## Funding

The authors have nothing to report.

## Ethics Statement

The authors have nothing to report.

## Consent

Written informed consent was obtained from the patient for publication of this case report and any accompanying images.

## Conflicts of Interest

The authors declare no conflicts of interest.

## Data Availability

The authors have nothing to report.
